# In‐loop flow [^11^C]CO_2_ fixation and radiosynthesis of *N*,*N*′‐[^11^C]dibenzylurea

**DOI:** 10.1002/jlcr.3568

**Published:** 2017-12-22

**Authors:** Joseph Downey, Salvatore Bongarzone, Stefan Hader, Antony D. Gee

**Affiliations:** ^1^ Division of Imaging Sciences and Biomedical Engineering King's College London London UK

## Abstract

Cyclotron‐produced carbon‐11 is a highly valuable radionuclide for the production of positron emission tomography (PET) radiotracers. It is typically produced as relatively unreactive carbon‐11 carbon dioxide ([^11^C]CO_2_), which is most commonly converted into a more reactive precursor for synthesis of PET radiotracers.

The development of [^11^C]CO_2_ fixation methods has more recently enabled the direct radiolabelling of a diverse array of structures directly from [^11^C]CO_2_, and the advantages afforded by the use of a loop‐based system used in ^11^C‐methylation and ^11^C‐carboxylation reactions inspired us to apply the [^11^C]CO_2_ fixation “in‐loop.” In this work, we developed and investigated a new ethylene tetrafluoroethylene (ETFE) loop‐based [^11^C]CO_2_ fixation method, enabling the fast and efficient, direct‐from‐cyclotron, in‐loop trapping of [^11^C]CO_2_ using mixed DBU/amine solutions. An optimised protocol was integrated into a proof‐of‐concept in‐loop flow radiosynthesis of N,N′‐[^11^C]dibenzylurea. This reaction exhibited an average 78% trapping efficiency and a crude radiochemical purity of 83% (determined by radio‐HPLC), giving an overall nonisolated radiochemical yield of 72% (decay‐corrected) within just 3 minutes from end of bombardment.

This proof‐of‐concept reaction has demonstrated that efficient [^11^C]CO_2_ fixation can be achieved in a low‐volume (150 μL) ETFE loop and that this can be easily integrated into a rapid in‐loop flow radiosynthesis of carbon‐11–labelled products.

This new in‐loop methodology will allow fast radiolabelling reactions to be performed using cheap/disposable ETFE tubing setup (ideal for good manufacturing practice production) thereby contributing to the widespread usage of [^11^C]CO_2_ trapping/fixation reactions for the production of PET radiotracers.

## 1 INTRODUCTION

One of the most prevalent and important radionuclides used in positron emission tomography (PET) is carbon‐11 (^11^C, t_1/2_ = 20.4 min); it has been incorporated into a wide variety of exogenous and endogenous ligands, used for both diagnostic and research purposes.^1^, ^2^, ^3^, ^4^, ^5^ The short half‐life potentially allows the administration and scanning of multiple PET radiotracers in the same patient on the same day, thereby combining the advantages of imaging multiple biochemical pathways; in addition, it enables the labelling of biologically relevant molecules, without changing their pharmacodynamic and pharmacokinetic properties, by virtue of its isotopology with carbon‐12.

Carbon‐11 is produced as [^11^C]CO_2_ (primary precursor), by the cyclotron proton‐bombardment of nitrogen‐14 via the ^14^N(p,α)^11^C nuclear reaction. The relatively low reactivity and solubility of [^11^C]CO_2_ leads to its rapid conversion to more reactive secondary precursors.^1^ The most prevalent of these secondary precursors is [^11^C]CH_3_I for ^11^C‐methylation reactions.^1^ While these reactions are used to produce the vast majority of carbon‐11 radiotracers, in the time taken to convert [^11^C]CO_2_ to [^11^C]CH_3_I, significant amounts of the starting radioactivity can be lost through multistep synthesis and radioactive decay.^6^ In addition, ^11^C‐methylation can somewhat limit the chemical space available for radiolabelling. As such, there have been a variety of alternative secondary and tertiary ^11^C‐precursors developed to convert the poorly reactive [^11^C]CO_2_ into a more versatile toolbox for the ^11^C‐radiochemist: [^11^C]CO, [^11^C]HCN, [^11^C]CS_2_, [^11^C]CH_3_OTf, and [^11^C]COCl_2_ are just a few examples.^1^

In an effort to avoid this time‐consuming conversion to more reactive precursors, [^11^C]CO_2_ has been reacted directly with Grignard reagents (alkyl magnesium bromide) to synthesise ^11^C‐carboxylic acid derivatives such as [^11^C]acetate, [^11^C]palmitic acid, and 2‐[^11^C]octynoic acid.^7^, ^8^, ^9^ In an effort to easily automate these syntheses, these reactions have been performed “in‐loop” via a captive solvent system; whereby the walls of a small tubing loop are coated with the highly reactive Grignard reagents, then [^11^C]CO_2_ is flowed through the loop, where it reacts in‐loop to form the ^11^C‐labelled carboxylic acid product.^10^, ^11^ This in‐loop synthetic approach has also been used in ^11^C‐methylation reactions using gaseous [^11^C]CH_3_I, with the synthesis of [^11^C]raclopride as a notable example.^12^, ^13^, ^14^, ^15^, ^16^ This synthetic methodology lends itself particularly well to the specific demands of ^11^C‐radiochemistry, and it has a number of advantages over the standard vial‐based methods. For these mixed gas‐liquid phase reactions, the high surface area provided by the walls of the loop maximises gas‐liquid contact, increasing the efficiency and thus decreasing the reaction times required versus the standard vial‐based setup. In addition, since loop synthesis eliminates reaction vials, transfer losses are kept to a minimum; and since this, in effect, miniaturises these reactions, precursor quantities can be greatly reduced. Finally, these reactions are easily automated, which is advantageous when considering the translational potential of a tracer.^12^, ^13^

More recently, there has been a resurgence of interest into the direct use of [^11^C]CO_2_ in radiolabelling using a technique called [^11^C]CO_2_ fixation.^6^, ^17^ This new chemistry is a by‐product of the “green‐chemistry” movement and the corresponding interest in new chemical agents for carbon‐capture;^6^, ^17^, ^18^, ^19^ the most commonly used compounds being the amidine base 1,8‐diazabicyclo[5.4.0]undec‐7‐ene (DBU),^20^ and the phosphazene base, 2‐tert‐butylimino‐2‐diethylamino‐1,3‐dimethylperhydro‐1,3,2‐diazaphosphorine (BEMP).^21^ While there is still some uncertainty regarding their trapping/fixation mechanisms,^22^, ^23^ solutions of these bases with an amine substrate exhibit efficient [^11^C]CO_2_ trapping and transfer to form amine carbamate intermediates.^20^ This [^11^C]CO_2_ fixation has therefore enabled many new ^11^C‐carbonylation reactions starting from [^11^C]CO_2_, and many new syntheses have been developed based on this fixation strategy (^11^C‐carbamates, ^11^C‐ureas, ^11^C‐amides, and ^11^C‐oxazolidinones, among others).^20^, ^24^, ^25^, ^26^, ^27^, ^28^, ^29^ Within our group, we have developed a Mitsunobu‐based method for the synthesis of ^11^C‐ureas.^26^, ^27^ This reaction involves the fixation of [^11^C]CO_2_ by DBU and amines to form the ^11^C‐amine carbamate intermediates; Mitsunobu reagents (PBu_3_/DBAD) are added to form an ^11^C‐isocyanate intermediate, which reacts with another amine molecule to form an ^11^C‐labelled urea product, in just 5‐minute total reaction time.^26^, ^27^

The recent interest in [^11^C]CO_2_ fixation chemistry has the potential to revolutionise the field of carbon‐11 radiochemistry, and the advantages afforded by the use of a loop‐based system used in ^11^C‐methylation and ^11^C‐carboxylation reactions inspired us to apply the [^11^C]CO_2_ fixation in‐loop.

In this work, our aim was therefore to (1) establish a novel in‐loop trapping/fixation of [^11^C]CO_2_ for the investigation and optimisation of different trapping solutions and (2) implement the setup in an in‐loop flow‐radiosynthesis of ^11^C‐labelled urea functional groups from [^11^C]CO_2_.

## 2 RESULTS AND DISCUSSION

### 2.1 Three‐component [^11^C]CO_2_ loop trapping

**Figure 1:**
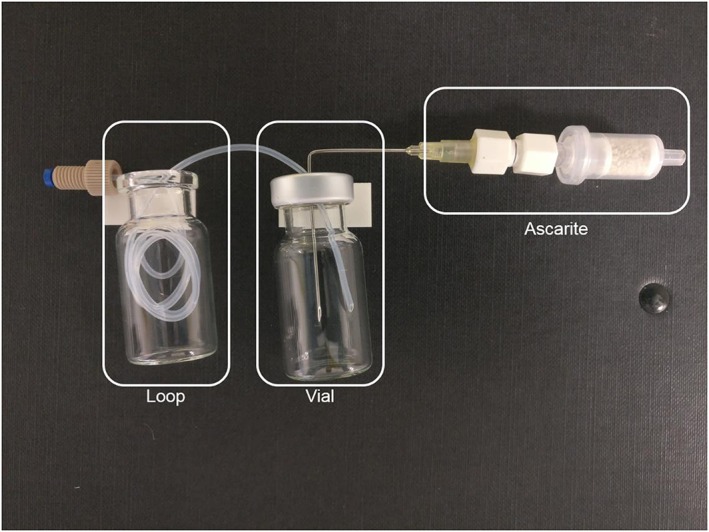
Representative 3‐component [^11^C]CO_2_ trapping: trapping loop, vial, and [^11^C]CO_2_ trapping cartridge (ascarite)

To assess the potential for in‐loop trapping/fixation of [^11^C]CO_2_, we designed a novel prototype 3‐component loop trapping apparatus (Figure 1). In designing the system, good manufacturing practice (GMP) requirements led us to opt for a single‐use disposable ethylene tetrafluoroethylene (ETFE)–based loop system since it obviates the need for rigorous cleaning etc. The system consists of 3 easily separable components: a trapping loop (1/16″ O.D. ETFE tubing, 150‐μL volume), the walls of which will be coated with a trapping solution; a crimped glass waste vial; and a trapping cartridge able to fix all unreacted [^11^C]CO_2_ (ascarite trap, Figure 1). On passing [^11^C]CO_2_ through this system, the [^11^C]CO_2_ is totally trapped within these 3 components. The components are then simply separated, and the radioactivity levels of the loop, waste vial, and ascarite trap (R_loop_, R_waste_, and R_trap_, respectively) are measured. Comparison of these values allows the calculation of trapping efficiencies for a given solution.

To precoat the walls of the loop, it is half filled with 75‐μL trapping solution, then it is connected to the cyclotron delivery line and flushed with helium gas through to the waste vial. The waste vial contains the trapping solution that was not retained by the loop. Therefore, when [^11^C]CO_2_ is passed through the 3‐component system, it is trapped within the loop and any untrapped [^11^C]CO_2_ will be fixed in the solution within the waste vial and the trapping cartridge.

To assess a solution's overall trapping efficiency as well as its suitability for our applications, we extracted 2 values from the acquired data: These are termed total‐solution trapping (T_sol_) and loop‐trapping (T_loop_).

T_sol_ (T_sol_ = (R_loop_ + R_waste_)/(R_loop_ + R_waste_ + R_trap_)) gives an insight into a solution's ability to trap [^11^C]CO_2_ in bulk and is a proxy measure for the chemical trapping efficiency of a given solution. While a low T_sol_ indicates that a solvent mixture is unsuitable for application within our in‐loop [^11^C]CO_2_ trapping/fixation method, a high T_sol_ does not conversely guarantee success for our work. Since a highly efficient trapping solution may still have poor loop‐retention, the degree to which the trapping solution is retained on the walls of the loop, and thus, [^11^C]CO_2_ trapping/fixation will not occur in‐loop but in the waste vial.

Instead T_loop_ (T_loop_ = R_loop_/(R_loop_ + R_waste_ + R_trap_)) accounts both for the chemical trapping ability of a solution, but also the degree to which it is retained within our 150‐μL ETFE loop. High values of T_sol_ and T_loop_ should guarantee success in developing an in‐loop [^11^C]CO_2_ fixation methodology.

Our model trapping solutions contained varying concentrations of benzylamine and DBU dissolved in acetonitrile (MeCN). The DBU was chosen instead of BEMP, since in previous work, using these Mitsunobu reagents, both compounds gave good [^11^C]CO_2_ trapping but only DBU led to ^11^C‐urea formation.^27^

To ensure MeCN does not exhibit any [^11^C]CO_2_ trapping itself, pure MeCN trapping experiments were attempted and – as expected considering the low solubility of [^11^C]CO_2_ in MeCN – negligible trapping was seen for this experiment (T_sol_ = 6.5 ± 0.1%, T_loop_ = 0.2%, *n* = 2).

**Figure 2:**
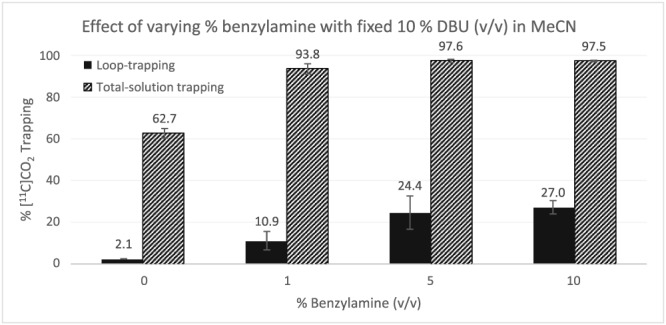
Effect of varying % benzylamine with fixed 10% DBU (*v*/v) in MeCN

Firstly, we explored the effect of varying benzylamine concentration (1%, 5%, and 10%) in the presence of a fixed content of DBU (10% *v*/v) (Figure 2). A solution of 10% DBU in MeCN without adding benzylamine (Figure 2) showed good T_sol_ (62.7 ± 2.3%), but very low T_loop_ (2.1 ± 0.1%). While the solution can trap [^11^C]CO_2_ reasonably well, it is poorly retained in the loop. Any solutions to be used within this loop trapping/fixation methodology must therefore exhibit high T_loop_. Increasing benzylamine content up to 10%, both T_sol_ and T_loop_ increased. Notably, adding 1%, 5%, and 10% benzylamine, T_sol_ showed near‐quantitative trapping of total [^11^C]CO_2_ (93.8 ± 2.5%, 97.6 ± 0.8%, and 97.5 ± 0.4%, respectively). However, despite the comparable T_sol_ seen for these solutions, there was a marked difference in T_loop_ values (Figure 2). Using 1% and 5% benzylamine content showed a two‐fold increase of T_loop_ (10.9 ± 4.4% and 24.4 ± 8.0%, respectively); however, increasing further the content of benzylamine up to 10% T_loop_ did not significantly improve (27.0 ± 3.2%).

These results show that—chemically—these mixed benzylamine/DBU solutions are exquisitely fine [^11^C]CO_2_ trapping/fixation solutions, even at relatively low concentrations of benzylamine (1%, T_sol_ > 90%). However, for their usage in loop trapping/fixation, only the solutions with high T_loop_ values are suitable (10% benzylamine, T_sol_ = 97%, T_loop_ = 27%). We suspected that the increased benzylamine content of the solutions with the highest T_loop_ values resulted in the increased viscosity of these solutions, replacing less‐viscous MeCN^30^ (0.343 mPa.s) with more viscous benzylamine^31^ (1.492 mPa.s), which therefore gave an increased retention of the solution on the walls of the loop.

**Figure 3:**
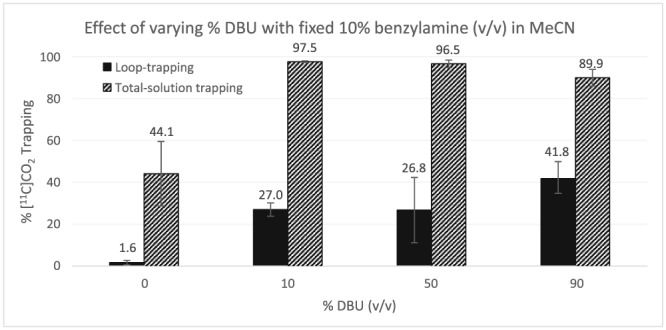
Effect of varying % DBU with fixed 10% benzylamine (v/v) in MeCN

Since we observed no significant improvement of T_loop_ using 5% or 10% of benzylamine, we decided to fix the content of benzylamine to 10% in the next experiments. We then explored the effect of varying DBU concentration in our solutions (Figure 3). In MeCN, 10% benzylamine (no DBU added) showed a reasonable T_sol_ (44.1 ± 15.4%), but very low T_loop_ (1.6 ± 1.1%). These results are very similar to those seen for a solution of 10% DBU in MeCN (Figure 2); indeed, while both solutions (10% DBU in MeCN or 10% benzylamine in MeCN) can trap [^11^C]CO_2_ fairly well, their poor loop‐retention hampers the T_loop_. Adding DBU at 10%, 50%, and 90% led to near‐quantitative T_sol_ (97.5 ± 0.4%, 96.5 ± 1.8%, and 89.9 ± 4.0%, respectively), again demonstrating that these are highly powerful [^11^C]CO_2_ trapping/fixation solutions (Figure 3). However T_loop_ increased by adding 10%, 50%, and 90% DBU (27.0 ± 3.2%, 26.8 ± 15.6%, and 41.8 ± 7.1, respectively). The highest T_loop_ value (42%) for this 3‐component trapping setup was obtained using an MeCN‐free system containing 90% DBU and 10% benzylamine (*v*/v) mixture. This again supports the suggestion that solution viscosity dictates loop‐retention and therefore affects T_loop_, since we are increasing the content of more viscous DBU^32^ (11.76 mPa.s) and decreasing the content of less viscous MeCN^31^ (0.343 mPa.s).

This section of work demonstrated the successful trapping and fixation of [^11^C]CO_2_ using a mixture of benzylamine/DBU solutions coating the walls of a 150‐μL ETFE loop. The modular 3‐component experimental setup was easy to set up as well as to disconnect for radioactivity measurements and so allows the rapid screening of a number of different trapping solutions. We next wanted to explore the potential integration of this in‐loop [^11^C]CO_2_ trapping into more complex flow syntheses of ^11^C‐labelled compounds. To investigate this, we performed a proof‐of‐concept flow synthesis of *N*,*N*′‐[^11^C]dibenzylurea using a more complex setup and using the optimal trapping solution developed above: 90% DBU and 10% benzylamine (*v*/v) mixture.

### 2.2 In‐loop flow radiosynthesis of *N*,*N′*‐[^11^C]dibenzylurea

**Figure 4:**
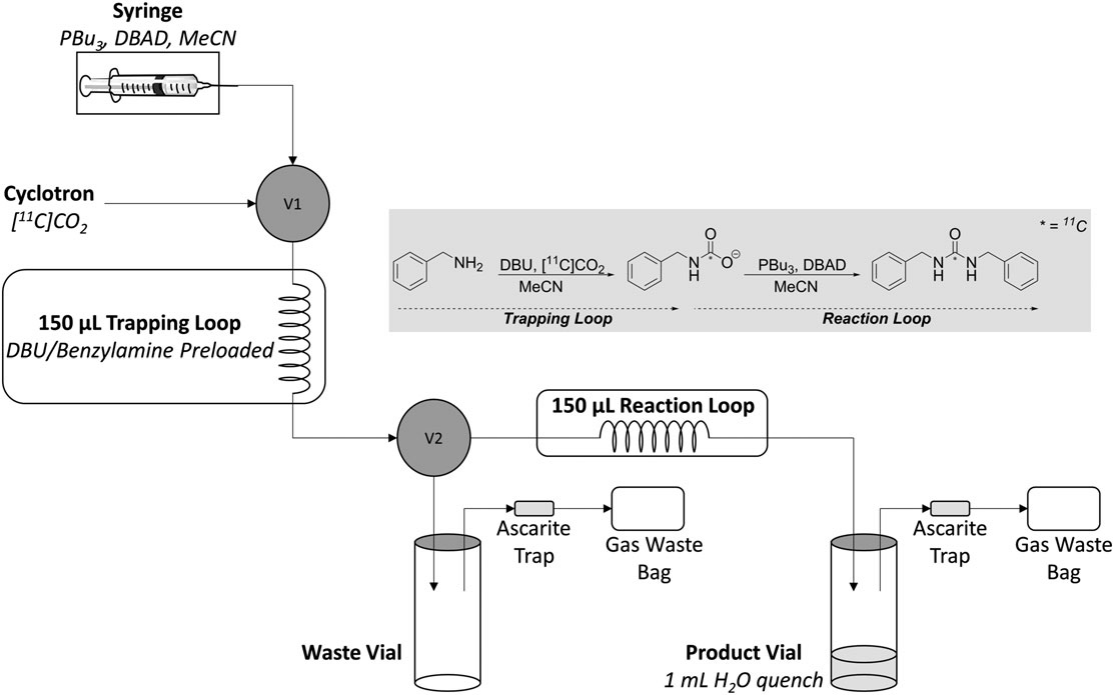
Schematic of in‐loop flow radiosynthetic setup showing the [^11^C]CO_2_ trapping loop as well as the additional reaction loop to allow Mitsunobu reaction to form *N*,*N*′‐[^11^C]dibenzylurea

The apparatus setup for the flow‐synthesis section of this work was based on the simple trapping apparatus described above. The [^11^C]CO_2_ trapping/fixation in‐loop was initially performed in a similar manner, before passing a solution of Mitsunobu reagents (PBu_3_ and DBAD in MeCN) through the trapping loop, through a second 150‐μL reaction loop (to ensure adequate mixing), and into a product vial (Figure 4). The [^11^C]CO_2_ is trapped initially as an *N*‐[^11^C]benzyl carbamate intermediate, which is converted by the Mitsunobu reagents to a highly reactive ^11^C‐isocyanate intermediate. This then undergoes attack from another molecule of benzylamine, to form the desired *N*,*N*′‐[^11^C]dibenzylurea product (Figure 4).

The setup (Figure 4) includes 2 switching valves (V1 and V2) on an E&Z Modular Lab automated synthesis system. The trapping loop is connected to these 2 valves, the inlet is connected to V1, and the outlet is connected to V2. V1 can be switched to either the cyclotron outlet (for helium flush and [^11^C]CO_2_ delivery) or a syringe containing the Mitsunobu reagents. V2 can divert the flow either towards the waste vial or through the reaction loop and into the product vial (see Section 4.1 for step‐by‐step procedure). To ascertain the quantity of [^11^C]CO_2_ delivered through the system per experiment, the system was washed with MeCN and the radioactivity of all the washings and components (lines, fittings, and needles) were measured.

An additional feature of this setup is the 1‐mL H_2_O added as a quenching solution in the final product vial. This ensures that any ^11^C‐urea derivatives detected in the crude HPLC are products formed exclusively in the loop. Without this simple addition, the reaction could feasibly be simply occurring in the product vial that receives the reaction mixture, during the time taken to measure product radioactivity and prepare the sample for HPLC analysis. The active Mitsunobu intermediate formed is a Morrison‐Brunn‐Huisgen betaine, which will react with protic substrates: alcohols, amines, and carboxylic acids.^33^, ^34^, ^35^, ^36^, ^37^ Our own non‐radioactive experiments have confirmed that this betaine reacts with water forming the tri‐*n*‐butylphosphine oxide (PBu_3_O) and di‐*tert*‐butyl hydrazodiformate (DBAD‐H_2_). Therefore, in the ^11^C‐urea synthesis, any remaining unreacted urea‐forming betaine will react with the excess water (in the product vial) upon leaving the reaction loop, ensuring that the crude‐HPLC is representative of the ^11^C‐labelled species formed in‐loop, not in‐vial. In addition, this serves to predilute the crude product ready for HPLC injection, which helps to streamline the synthetic process.

One notable observation during these ^11^C‐urea syntheses was that we saw a significantly increased T_loop_ (78.3 ± 3.6%) compared to that seen within the trapping experiments (41.8 ± 7.1%), indicating an increase in loop‐retention of our optimised trapping solution. This variance in T_loop_ value might be due to the difference between the 2 setups; in the flow synthesis of *N*,*N*′‐[^11^C]dibenzylurea, the loops are routed via the E&Z switching valves (V1 and V2). We speculate that the use of these valves increases the backpressure in the system and correspondingly increases the loop retention of the trapping solution.

Because of the demanding time‐constraints placed upon carbon‐11 radiochemistry in the development of this method, we attempted to minimise all process times, avoiding any product losses because of radioactive decay. Since we avoided pretrapping or concentration of [^11^C]CO_2_ (instead delivered diluted in the helium carrier gas), and the rate of delivery was not slowed from the cyclotron's 70 mL/min, the [^11^C]CO_2_ is delivered through the system and trapped in the loop within 105 seconds of the end of bombardment (EOB). V1 and V2 are instantly switched, and the trapping loop is filled with Mitsunobu reagents within 30 seconds (Figure 4). V1 is then switched back to a helium flush, and the reagents are pushed through the reaction loop and into the product vial at 70 mL/min. Therefore, the process is complete within just 3 minutes from the EOB and 1 minute from the end of delivery.

In the synthesis of our model substrate, *N*,*N*′‐[^11^C]dibenzylurea, we had to consider the concentration and stoichiometry of our Mitsunobu reagents added to the 150‐μL trapping loop. Based on the assumption of 7‐ to 8‐μL trapping solution retention (based on preliminary non‐radioactive flushing experiments filling the loop with 75 μL of solution) and considering the optimal conditions found in our previous radiosynthesis of ^11^C‐symmetrical ureas,^27^ the reagent concentration was selected to ensure a 2:1 stoichiometric ratio of Mitsunobu reagents (PBu_3_/DBAD) to benzylamine.

Using the in‐loop flow radiosynthesis setup, the radio‐HPLC of the crude solution showed a radiochemical purity of 82.6 ± 3.3%. This coupled with the overall T_loop_ of 78.3 ± 3.6% led to a decay‐corrected nonisolated radiochemical yield (RCY) of 72.3 ± 5.1% (n = 3) for the synthesis of *N*,*N*′‐[^11^C]dibenzylurea, in 3 minutes from EOB, with a molar radioactivity of 0.72 ± 0.17 GBq/μmol (for 300‐350 MBq initial [^11^C]CO_2_; other work in the group has demonstrated that this molar radioactivity would be expected to increase towards 60 to 70 GBq/μmol for an initial 30 GBq [^11^C]CO_2_ production,^28^ which is consistent with the molar radioactivities obtained for other clinical ^11^C‐labelled radiotracers within our institution).^38^ This therefore presents a reproducible, rapid, and high‐yielding synthesis of a carbon‐11–labelled urea product directly from [^11^C]CO_2_.

The RCYs for the synthesis of *N*,*N*′‐[^11^C]dibenzylurea using this in‐loop method (72% decay‐corrected) are slightly lower than those for the traditional in‐vial method (82% decay‐corrected), because of a lower [^11^C]CO_2_ trapping efficiency (78% vs 96%), but with a comparable radiochemical purity (83% vs 85%, by crude radio‐HPLC).^27^ However, this in‐loop method uses smaller quantities of reagents compared to the in‐vial method (75 μL vs 400 μL), which should simplify purification, and will minimise most of the transfer losses associated with in‐vial synthesis, and the use of cheap, disposable, ETFE loops means that this method is particularly well suited to GMP production. These factors combined therefore mean that this in‐loop method presents an appealing alternative to in‐vial [^11^C]CO_2_ fixation reactions.

## 3 CONCLUSIONS

In this work, we demonstrated that cyclotron‐produced [^11^C]CO_2_ can be fixed in a low‐volume (150 μL) ETFE loop. We showed that while amine/DBU solutions are chemically efficient [^11^C]CO_2_ fixation agents in‐vial, both the chemical and physical properties (primarily viscosity) of these solutions determine their degree of loop trapping efficiency (T_loop_). This optimised direct‐from‐cyclotron [^11^C]CO_2_ fixation methodology avoids the need for cryogenic preconcentration commonly used in carbon‐11 procedures. This setup was implemented in a proof‐of‐concept in‐loop flow radiosynthesis of *N*,*N*′‐[^11^C]dibenzylurea by passing Mitsunobu reagents through the loop containing a trapped *N*‐[^11^C]benzyl carbamate intermediate and benzylamine. *N*,*N*′‐[^11^C]dibenzylurea was obtained with high nonisolated RCYs (approximately 72%), comparable to those previously reported in‐vial (approximately 82%), within 3 minutes from EOB (recently presented in abstract form at the International Symposium on Radiopharmaceutical Sciences, ISRS, Dresden, 14‐19 May 2017).^39^ This novel methodology has demonstrated the potential for direct‐from‐cyclotron in‐loop [^11^C]CO_2_ fixation and has demonstrated that this can be used as part of a more complex synthesis (eg, amides and carbamates).^28^ The speed of the reaction (3 min from EOB) and the cheap/disposable ETFE tubing setup (ideal for GMP production) mean that this method should be suitable for further applications in the direct trapping/fixation reactions of [^11^C]CO_2_. We anticipate that this new methodology will facilitate a more widespread uptake and application of these powerful reactions, for the radiolabelling of a diverse array of structures directly from [^11^C]CO_2_.

## 4 EXPERIMENTAL

### 4.1 Materials and general methods

Anhydrous acetonitrile (MeCN, 99.8%), ascarite, benzylamine (99%), di‐*tert*‐butyl‐azodicarboxylate (DBAD, 98%), triethylamine (Et_3_N, ≥99.5%), and tri‐*n*‐butyl phosphine (PBu_3_, 99%) were purchased from Sigma‐Aldrich. Ethyl acetate (EtOAc, ≥99.5%) was purchased from Fisher Scientific. Anhydrous magnesium sulphate (MgSO_4_, 98%) was purchased from Fluka. 1,8‐Diazabicyclo[5.4.0]undec‐7‐ene (DBU, 99%) and benzyl isocyanate (98%) were purchased from Alfa Aesar. Carbon dioxide (CO_2_) was purchased from BOC Gases.

The ETFE tubing (1/16″ O.D. × 0.75 mm I.D., 25 m/pkg) was obtained from VICI Jours. The ascarite traps were constructed from empty SPE‐ED cartridges, obtained from Biosys Solutions Ltd: Fritted Empty MiniSPE‐ED Cartridges, part # 2447. All fluidic connections were obtained from Upchurch Scientific; the product codes are as follows: fingertight flangeless fitting short, PEEK, XP‐235X; female to male quick‐connect Luer adapter, P‐675‐01.

[^11^C]CO_2_ was produced using a Siemens RDS112 cyclotron in a ^14^N(p,α)^11^C reaction, by the 11‐MeV proton bombardment of nitrogen (+1% O_2_) gas. The cyclotron produced [^11^C]CO_2_ was transferred in a stream of helium gas at 70 mL/min directly into a switching valve of an E&Z Modular Lab automated synthesis unit. Unless otherwise specified, all radioactive experiments used a 5‐μA bombardment for 1 minute, giving on average 300 to 350 MBq [^11^C]CO_2_ at EOB. The RCYs reported are calculated as a percentage of the total radioactivity delivered from the cyclotron. Unless otherwise stated, all experiments were repeated 3 times (n = 3).

^1^H and ^13^C{^1^H} NMR spectra were recorded on a Bruker Avance DRX 400 MHz spectrometer at 294 K. Chemical shifts are reported in parts per million (ppm) relative to the residual solvent proton impurities (^1^H) or the residual solvent carbon impurities (^13^C), as internal standards.

The HPLC analysis was performed on an Agilent 1200 system, with a variable wavelength UV detector and a LabLogic Flow‐RAM β^+^ detector equipped in series. Analytical reverse‐phase column: Agilent XDB‐C18, 5 μm, 4.6 × 150 mm. Gradient used: 95% H_2_O, 5% MeCN; to 5% H_2_O, 95% MeCN; over 9 minutes. Identity of radioactive products was confirmed by coelution with the non‐radioactive standard compounds. The HPLC was used to determine molar radioactivities, by reference to a variable dilution calibration curve. Previous experiments within our laboratory have shown that scaling reactions to higher starting radioactivities, with all other factors kept constant, lead to correspondingly higher molar radioactivities. From these results, we assume that a 100‐fold increase from approximately 300 MBq (preliminary experiments) to 30 GBq initial [^11^C]CO_2_ (clinical production) will give a roughly 100‐fold increase in molar radioactivity.

Mitsunobu solutions were prepared by dissolving DBAD (21.1 mg, 91.6 μmol, 3 eq.) in 1‐mL MeCN (anhydrous). PBu_3_ (22.9 μL, 91.6 μmol, 3 eq.) was added, and the mixture was briefly shaken. A colour change from pale yellow to colourless was observed on successful formation of the active Mitsunobu intermediate. Trapping solutions were prepared as 1‐mL solutions by diluting the corresponding percentages (*v*/v) of DBU and benzylamine in MeCN. All trapping (benzylamine/DBU/MeCN) and Mitsunobu (PBu_3_/DBAD/MeCN) solutions were prepared under an inert argon atmosphere, using anhydrous MeCN.

### 4.2 Three‐component [^11^C]CO_2_ trapping apparatus: design and setup

The trapping apparatus setup is shown in Figure 1. One end of a 35‐cm length of ETFE tubing (trapping loop, 1/16″ O.D., 0.75 mm I.D., 150‐μL volume) was fitted with a fingertight screw fitting, and the other end was cleanly cut at a 45° taper. The loop was tightly coiled and placed inside a 10‐mL glass vial for ease of handling. The tapered end was inserted through the rubber septum of a sealed crimped 10‐mL waste vial. This vial was vented via a needle through an ascarite trap into a gas waste bag. The loop was half filled with 75‐μL trapping solution, using a 100‐μL syringe, and connected via a Luer slip fitting to the [^11^C]CO_2_ outlet line from the E&Z Modular Lab, providing a simple, modular, and easy to disconnect setup, which traps all [^11^C]CO_2_ passed through.

### 4.3 ^11^C‐urea derivative flow synthesis apparatus: design and setup

The ^11^C‐urea synthesis apparatus builds upon the trapping apparatus proof‐of‐concept and is shown in Figure 4. The cyclotron [^11^C]CO_2_ outlet line was connected to switching valve 1 (V1) as was a syringe containing Mitsunobu reagents (PBu_3_ and DBAD in MeCN). Both ends of a 35‐cm length of ETFE tubing (trapping loop, 1/16″ O.D., 0.75 mm I.D., 150‐μL volume) were fitted with fingertight screw fittings, and the loop was half filled with 75‐μL trapping solution. One end was attached to the outlet of V1 and the other to the inlet of switching valve 2 (V2). To one outlet of V2, a short length of ETFE tubing was connected to a waste vial, vented via an ascarite trap. To the other outlet is connected a second 35‐cm length of coiled ETFE tubing (reaction loop, 1/16″ O.D., 0.75 mm I.D., 150 μL) running into a sealed crimped product vial (containing 1‐mL water), vented via an ascarite trap.

### 4.4 Generic procedure: [^11^C]CO_2_ trapping

The preloaded loop was connected to the [^11^C]CO_2_ delivery line from the cyclotron, and helium was flushed through the system for 3 minutes at 70 mL/min, leaving a just a small, residual amount of trapping solution coating the walls of the trapping loop, and flushing the bulk of the trapping solution into the waste vial. The cyclotron produced [^11^C]CO_2_ was then directly delivered diluted in carrier helium gas (without prior trapping and concentration) at 70 mL/min into the E&Z Modular Lab and subsequently through the 3‐component trapping apparatus. Since all [^11^C]CO_2_ is trapped within either the trapping loop, the waste vial, or the ascarite trap, these 3 components are quickly separated and their radioactivities measured within a Capintec dose calibrator. Comparison of the distribution of radioactivity within this apparatus allowed calculation of different solvent trapping efficiencies.

### 4.5 Generic procedure: ^11^C‐urea derivative synthesis

(1) Helium was flushed through the preloaded trapping loop to the waste vial for 3 minutes at 70 mL/min. (2) Cyclotron produced [^11^C]CO_2_ was then directly delivered, diluted in carrier helium gas (without prior trapping and concentration) at 70 mL/min, through the trapping loop, waste vial, and ascarite trap. (3) V1 and V2 are switched, and the trapping loop was filled with 150‐μL Mitsunobu solution (PBu_3_/DBAD in MeCN). (4) V1 was switched, and a 70‐mL/min helium flush from the cyclotron transferred the contents of the trapping loop through the reaction loop and into the product vial (containing 1‐mL water as a quench). (5) The crude products were analysed by radio‐HPLC and the system was washed with MeCN, and all washings and component radioactivities were measured to determine the total [^11^C]CO_2_ radioactivity delivered from the cyclotron.

### 4.6 Synthesis of *N*,*N*′‐dibenzylurea

Benzylamine (1.2 mmol, 131 μL, 3 eq.) was dissolved in 2‐mL EtOAc with stirring at room temperature. To this was added Et_3_N (1.2 mmol, 167 μL, 3 eq.) and benzyl isocyanate (400 μmol, 49.4 μL, 1 eq.). Solution was stirred for 2 hours under ambient conditions. 1 M HCl was added, and the solution was extracted 3 times with EtOAc. The combined organic extracts were washed with brine and dried over MgSO_4_. The solvent was removed under reduced pressure to yield a white solid (83.5 mg, 87% yield); ^1^H NMR (400 MHz, CDCl_3_) δ 7.25‐7.1 (m, 10H, CH), 4.75 (br s, 2H, NH), 4.26 (d, 4H, CH_2_); ^13^C NMR (100 MHz, CDCl_3_) δ 158.1 (CO), 139.0 (C), 128.6 (CH), 127.4 (CH), 127.3 (CH), 44.5 (CH_2_).
